# PBC: Animal Models of Cholangiopathies and Possible Endogenous Viral Infections

**DOI:** 10.1155/2012/649290

**Published:** 2011-08-08

**Authors:** Masashi Ninomiya, Yoshiyuki Ueno, Tooru Shimosegawa

**Affiliations:** Division of Gastroenterology, Department of Gastroenterology, Tohoku University Graduate School of Medicine, Seiryo, Aoba-ku, Sendai 980-8575, Japan

## Abstract

Primary Biliary Cirrhosis (PBC) is considered an autoimmune disease characterized by immune-mediated destruction of the intrahepatic bile ducts and its characteristic serologic marker, the anti-mitochondrial antibody (AMA). Several factors were proposed to clarify the pathological and immunological mechanisms of PBC. Immunological reaction with a bacterial or a viral association was identified in the previous report, and it seems probable that PBC was thought to have such an etiology. The majority of patients with PBC was reported to have both RT-PCR and immunohistochemistry evidence of human betaretrovirus infection in lymph nodes or in 2008, the patient who developed PBC with high HIV viral load had an antiviral therapy and recovered. To understand the etiology of PBC associated with infection, several factors should be considered and especially animal models may be useful. In this paper, we introduce three typical animal models of PBC: the dominant-negative form of transforming growth factor-**β** receptor type II (dnTGF**β**RII) mouse, IL-2R**α** ^−/−^ mouse and NOD.c3c4 mouse, are enumerated and described, and we discuss previous reports of viral infection associated with PBC and consider the etiology of PBC from our analysis of results in NOD.c3c4 mouse.

## 1. Introduction

Primary Biliary Cirrhosis (PBC) is considered an autoimmune disease characterized by immune-mediated destruction of the intrahepatic bile ducts and its characteristic serologic marker, the anti-mitochondrial antibody (AMA). AMA is a highly specific autoantibody found in about 90% of patients with PBC that reacts with an epitope on the E2 subunit of the pyruvate dehydrogenase enzyme complex (PDC-E2) [1–3]. The epitopes discerned by anti-PDC-E2 and CD4 and CD8 autoreactive T cells are present in the inner lipoyl domain of PDC-E2. A 100-fold increase in CD4 and a 10-fold increase in CD8 autoreactive T cells infiltrate into the portal tracts [4, 5]. Moreover, several factors were proposed to clarify the pathological and immunological mechanisms of PBC. Some biological features of the bile duct cells have been reported, suggesting a basis for their distinctive destruction [6–8]. Optionally, soon after autoimmune diseases were first recognized more than a century ago, immunological reaction with a bacterial or a viral association was identified and PBC was thought to have such an etiology (Table 1). [9–11]. The majority of patients with PBC were reported to have both RT-PCR and immunohistochemistry evidence of human betaretrovirus infection in lymph nodes [12], or in 2008, the patient who developed PBC with high HIV viral load had an antiviral therapy and recovered [13]. To determine whether PBC can be induced by infections, first autoimmunity needs to be defined. Autoimmune diseases occur when a response to a self-antigen involving T cells, B cells, or autoantibodies induces injury systemically or against a specific organ [14]. Although an autoimmune response occurs in most persons, it is only in a few persons that disease actually appears. In PBC, how can infection induce autoimmunity? The mechanism to explain the association of infection is molecular mimicry of autoepitops by peptides of microorganisms. This results in cryptic T-cell epitopes, the degeneracy of T-cell receptors, and the disruption of immune tolerance [15, 16]. This is of great significance for PBC because of the tendency of several viruses to target particularly the liver. There are several mechanisms by which viruses are thought to induce an autoimmune response. These include the expression of some autoantigens, the expression of major histocompatibility complex molecules, and changes in cytokine production [16]. To understand the etiology of PBC associated with infection, several factors should be considered and especially animal models may be useful [14, 17]. The association of betaretroviral protein production and aberrant PDC-E2-like protein expression in the IL-2R*α* ^−/−^ mouse and Nonobese diabetic (NOD).c3c4 mouse was reported recently [18].

 In this paper, we introduce three typical animal models of PBC: the dominant-negative form of transforming growth factor-*β* receptor type II (dnTGF*β*RII) mouse, IL-2R*α* ^−/−^ mouse, and NOD.c3c4 mouse are enumerated and described [19–21]. Additionally, we discuss previous reports of viral infection associated with PBC and consider the etiology of PBC from our analysis of results in NOD.c3c4 mouse.

## 2. Murine Model of PBC

### 2.1. DnTGF*β*RII Mouse

 TGF-*β* is the most widely distributed cytokine with pleiotropic effects on cell growth and immunological controls, specifically having a promoting effect on the development of the regulatory T-cell compartment [22]. dnTGF*β*RII mice were originally developed by Gorelik and Flavell for the purpose of analyzing the role of this receptor, which regulates the activation of the T cell function [23]. To disrupt the intracellular domain of the normal receptor in this mouse, the receptor is incompetent of transduction after TGF-*β* ligation. The expression of dnTGF*β*RII is limited by the CD4 promotor which lacks CD8 silencer, and this transgenic mouse spontaneously develops features characteristic of PBC [23]. These features include the expression of AMA with specificity against PDC-E2, BCOADC-E2, and OGDC-E2, as in human PBC. Pathologically, the infiltration of lymphoid cells, especially CD4^+^ and CD8^+^ lymphocytes, in the portal tracts causes biliary duct destruction [19] and the accumulation of natural killer T cells (NKT) in the intrahepatic bile duct lesions, resembling the condition found in human PBC [24]. Although the granuloma formations around the portal tracts seen in human PBC are not present, some lymphocytic aggregations like immature granuloma formation could be observed [25]. Furthermore, the serum levels of cytokines such as IFN-*γ*, TNF-*α*, IL-12p40, and IL-6 are significantly increased, as seen in human PBC [26, 27].

### 2.2. IL-2R*α* ^−/−^ Mouse

 In 2006, Aoki et al. reported a male child with a genetic deficiency of IL-2 receptor *α* IL-2R*α*, )CD25) expression who had liver dysfunction with serological expression of PBC. Histologically, there was lymphoid infiltration in the portal tracts and serum antibody to PDC-E2. The deficiency of CD4^+^ CD25^+^ subset of regulatory T cells was considered a key to elucidating of this clinical condition [20]. Based on these findings, Wakabayashi et al. established IL-2R*α* ^−/−^ mice and evaluated their hepatic immunopathology [28]. These mice also show AMA positivity against PDC-E2 that localizes to the inner lipoyl domain of the autoantigen. Lymphoid cells, composed of CD4^+^ and CD8^+^ lymphocytes, infiltrate into portal tracts without a significant increase in NKT. Although mild interface hepatitis and biliary duct destruction are seen in the liver, granuloma formations around the portal tracts are not observed [28]. The circulating cytokine profiles are similar to those of dnTGF*β*RII mice, showing elevations of IFN-*γ*, TNF-*α*, IL-12p40, and IL-6, as identified in the serum of patients with PBC [26, 27, 29]. 

### 2.3. NOD.c3c4 Mouse

 NOD.c3c4 mice were generated by the introgression of large genetic intervals on chromosome 3 and 4 into a NOD background [21, 30]. NOD and genetically modified NOD mice have been reported to progress to not only spontaneous autoimmune diabetes but also rheumatoid arthritis, Sjogren's syndrome, and thyroiditis [31–34]. NOD.c3c4 mice derived from NOD strains are considered to be an animal model of PBC with autoimmune biliary destruction [21, 30]. Most importantly, these mice show antibodies to PDC-E2. They express AMA positivity, unlike the dnTGF*β*RII mice and IL-2R*α* ^−/−^ mice, and the rate of positivity has reached 50–60% [35]. Portal tract infiltration with CD3^+^, CD4^+^, and CD8^+^ lymphocytes results in chronic nonsuppurative destructive cholangitis and epithelioid granuloma formations [21, 30]. However, the morphological features of the bile ducts lesions differ from those in human PBC, in which characteristic biliary cyst formations as well as apparent hepatomegaly are described [36].

## 3. Possibility of Viral Infection Associated with PBC

 It has been thought that some viruses may associate with human diseases of oncogenesis or autoimmunity because of their genome integration or specific viral-encoding proteins. Especially, in 1998, Munoz et al. described that there was an antibody for human immunodeficiency virus-1 (HIV-1) in the serum of PBC patients [37]. To investigate for a possible immune response to the p24 gag protein of HIV-1, moderate-to-strong reactivity was found in about 30% of the patients with Sjogren's syndrome, as compared with less than 1% of healthy controls [38], and the 36% of systemic lupus erythematosus (SLE) patients produced antibodies to the p24 gag protein [39]. Mason et al. discovered HIV-1 p24 gag protein seroreactivity in 35% of patients with PBC, 29% of patients with SLE, and 39% of patients with either primary sclerosing cholangitis or biliary atresia, compared with only 4% of patients with alcohol-related liver disease or alpha1-antitrypsin-deficiency liver disease, and only 4% of healthy volunteers. Moreover, Western blot reactivity to the human intracisternal A-type particle (HIAP) proteins related to HIV-1 was found in 51% of patients with PBC, in 58% patients with SLE, and in 17% of those with other biliary diseases. None of the 23 patients with either alcohol-related liver disease or alpha1-antitrypsin deficiency and only one of the healthy controls showed the same reactivity to HIAP proteins [40]. Therefore, these antibody reactivities found in patients with PBC may be attributable to an immune response to uncharacterized viral proteins that share antigenic determinants with HIV-1-related retroviruses.

 In 2003, a human betaretrovirus clone sequence was originally detected from the biliary epithelium cDNA library of a patient with PBC. When searching viral data registered in BLASTN, the initial partial pol gene fragment was found to exhibit 95% to 97% identities with mouse mammary tumor virus (MMTV) and with retrovirus sequences derived from human breast cancer samples within the overlapping sequence [12, 41]. Using a specific MMTV antibody, viral proteins were shown in the perihepatic lymph nodes but not in liver tissue samples from patients with PBC [41]. However, Selmi et al. expressed an opposing view concerning this result [42]. 

 Some pilot studies were conducted to determine whether antiviral therapy impacted the disease progression (Table 2). First, Mason et al. performed a trial with reverse-transcriptase inhibitors (lamivudine group versus lamivudine/zidovudine group) for patients with PBC. The lamivudine/zidovudine group showed significant serological improvement in the activities of alkaline phosphatase, AST and ALT, and histological assessment revealed an improvement in the necroinflammatory score and a reduction in bile duct injury compared to the lamivudine group [43]. A further clinical trial was performed with a combination of lamivudine and zidovudine versus ursodeoxycholic acid (UDCA). Significant differences were observed in the antiviral therapy versus UDCA with serological improvements in serial alkaline phosphatase, ALT and AST as well as the clinical score [44]. Thus, reverse-transcriptase inhibitors are expected to suppress retroviral proliferation and contribute to the improvement of PBC. We once investigated the efficacies of 90 day's administration of lamivudine to 20 PBC patients with unsatisfactory biochemical responses to UDCA in a randomized double blind control trial. As a result, no significant biochemical difference was seen between both groups. However, of interest, one case showed a decrease of AMA titers and biochemical response [45]. These results were similar to those of studies conducted by Mason et al. [43]; yet the true efficacy should be evaluated in large-scale control trials.

## 4. Are NOD.c3c4 Mice Infected with a Retrovirus?

 NOD.c3c4 mice have been described as a mouse model with several features similar to PBC [30]. However, these mice develop marked biliary cyst formation that is not shown in human PBC. In human PBC, the destruction of cholangiocytes leads to ductpenia [48]. When we analyzed the gene expression of the cholangiocytes of such mice using microarray analysis, there was consistent liver-specific downregulation in the expression of Fas antigen (CD95) [36]. Fas (CD95) antigen is a member of the tumor necrosis factor family that binds to Fas ligand (FasL). This gene is situated at chromosome 19 in mouse [49]. Fas/FasL interactions play an important role in apoptosis [49–51]. Fas is detected in hepatocytes and plays an important role in inflammation and cell death in hepatitis virus-infected liver [52]. The Fas system on cholangiocytes has been also reported in human and rats, and enhanced expression of FasL on cholangiocytes has been implicated in progressive bile duct loss in PBC through apoptosis [53]. Furthermore, FasL expressed by cholangiocarcinomas was reported to induce lymphocyte cell death and escape immune surveillance [54]. It has been reported that the Fas/FasL system is strongly associated with biliary pathological conditions. It was thought that, because of the downregulation of Fas antigen, the apoptosis of cholangiocytes cannot easily occur and therefore biliary cyst formation was found in NOD.c3c4 mice [36]. However, it was considered that there are also other factors because the degree of the cyst formation differed according to the individual though the genetic expression was the same.

 In 2007, Chen et al. reported that MMTV gag and pol gene expression was 4- to 25-fold higher in all three autoimmune biliary disease models, NOD.c3c4, dnTGF-*β*RII, and IL-2R*α* ^−/−^, as compared to control mice when using real-time RT-PCR to quantify MMTV using gag and pol primers [55]. A randomized study was conducted using NOD.c3c4 mice treated with a combination therapy of lamivudine and zidovudine or with placebo (Table 2). Serial hepatic biochemistry studies showed diminished alkaline phosphatase in the mice treated with combination therapy. Histological evaluation showed a significant decrease in the necroinflammatory score and the grade of the bile duct injury in the combination therapy group. However, therapy had little improvement on bile duct cyst formation. When compared to mice receiving placebo group, combination therapy group reduced viral burden measured by the pol and gag gene RT-PCR [46]. Moreover, Graham et al. treated NOD.c3c4 mice with combination of reverse transcriptase inhibitors and protease inhibitor. Hepatic biochemistry showed significant improvements in alkaline phosphatase and AST, and the cholangitis completely disappeared [47]. Thus, the detection of MMTV in the NOD.c3c4 mice and resolution of biliary disease with antiviral therapy supports the retroviral hypothesis for PBC.

 However, in order to prove that viral factors are involved in the pathogenesis of PBC, it is necessary to understand and elucidate the mechanism of viral replication, reproduction, the transcription of the virus genomes, the pathogenic roles of viral tropics, the integrated state, and the interaction with the host's immune system, especially the mechanism by which autoimmune tolerance is broken. Therefore, it is premature to relate the cause of the clinical condition of PBC to viral infection based only on the present report. However, given the unprecedented progress of biotechnology, a more detailed understanding of these issues can be expected in the near future.

## Figures and Tables

**Table 1:** Viral infections in humans associated with autoimmune diseases.

Relevance or suspicion of autoimmune human diseases	Representative viruses
PBC	HIV-1 p24
	MMTV
Multiple sclerosis	Epstein-Barr virus (EBV) Measles virus
Type1 diabetes	Coxsackie virus B4
	Rubella virus
	Cytomegalovirus (CMV) Mumps virus
Rheumatoid arthritis	EBV
	Hepatitis C virus (HCV)
Systemic lupus erythematosus	EBV
Myocarditis	Coxsackievirus B3
	CMV
Myasthenia gravis	Herpes simplex virus
	HCV
Guillain-Barre syndrome	CMV
	EBV

**Table 2:** Antiviral trials for PBC.

Trial	Method	Subject	Design	Primary outcome	Year	Reference
Pilot studies of single and combination antiretroviral therapy	Lamivudine + Zidovu- dine versus Lamivudine	Human	Randomized controlled trial (RCT)	Serological improvements of alkaline phos-phatase, AST and ALT. Histological improvement in necroinflammatory score and a reduction in bile duct injury.	2004	[43]
Clinical trial: randomized controlled trial of lamivudine and zidovudine (Combivir)	Lamivudine + Zidovu- dine + UDCA versus UDCA	Human	RCT	Serological improvements in serial alkaline phosphatase, ALT and AST.	2008	[44]
Randomized controlled trial of lamivudine	Lamivudine versus UDCA	Human	RCT	One case showed a decrease of AMA titers.	2010	[45]
Combination antiretroviral therapy with Combivir	Lamivudine + Zidovu- dine versus Placebo	NOD.c3c4 mouse		Histological improvement in necroinflammatory score and a reduction in bile duct injury. No improvement on bile duct cyst. Decrease in viral burden.	2007	[46]
Highly active antiretroviral therapy with reverse trans- criptase inhibitors and protease inhibitor	Combination of reverse transcriptase and protease inhibitor	NOD.c3c4 mouse		Serological improvements in alkaline phos-phatase and AST. Complete disappearance of cholangitis.	2008	[47]

## References

[1] Nakanuma Y, Ohta G (1979). Histometric and serial section observations of the intrahepatic bile ducts in primary biliary cirrhosis. *Gastroenterology*.

[2] Gershwin ME, Mackay IR, Sturgess A, Coppel RL (1987). Identification and specificity of a cDNA encoding the 70 KD mitochondrial antigen recognized in primary biliary cirrhosis. *Journal of Immunology*.

[3] Coppel RL, McNeilage LJ, Surh CD (1988). Primary structure of the human M2 mitochondrial autoantigen of primary biliary cirrhosis: dihydrolipoamide acetyltransferase. *Proceedings of the National Academy of Sciences of the United States of America*.

[4] van de Water J, Ansari AA, Surh CD (1991). Evidence for the targeting by 2-oxo-dehydrogenase enzymes in the T cell response of primary biliary cirrhosis. *Journal of Immunology*.

[5] Jones DEJ, Palmer JM, James OFW, Yeaman SJ, Bassendine MF, Diamond AG (1995). T-cell responses to the components of pyruvate dehydrogenase complex in primary biliary cirrhosis. *Hepatology*.

[6] Harada K, Ozaki S, Gershwin ME, Nakanuma Y (1997). Enhanced apoptosis relates to bile duct loss in primary biliary cirrhosis. *Hepatology*.

[7] Odin JA, Huebert RC, Casciola-Rosen L, LaRusso NF, Rosen A (2001). Bcl-2-dependent oxidation of pyruvate dehydrogenase-E2, a primary biliary cirrhosis autoantigen, during apoptosis. *Journal of Clinical Investigation*.

[8] Tanaka A, Leung PSC, Kenny TP (2001). Genomic analysis of differentially expressed genes in liver and biliary epithelial cells of patients with primary biliary cirrhosis. *Journal of Autoimmunity*.

[9] Fussey SPM, Ali ST, Guest JR, James OFW, Bassendine MF, Yeaman SJ (1990). Reactivity of primary biliary cirrhosis sera Escherichia coli dihydrolipoamide acetyltransferase (E2p): characterization of the main immunogenic region. *Proceedings of the National Academy of Sciences of the United States of America*.

[10] Long SA, Quan C, van de Water J (2001). Immunoreactivity of organic mimeotopes of the E2 component of pyruvate dehydrogenase: connecting xenobiotics with primary biliary cirrhosis. *Journal of Immunology*.

[11] Leung PSC, Quan C, Park O (2003). Immunization with a xenobiotic 6-bromohexanoate bovine serum albumin conjugate induces antimitochondrial antibodies. *Journal of Immunology*.

[12] Xu L, Shen Z, Guo L (2003). Does a betaretrovirus infection trigger primary biliary cirrhosis?. *Proceedings of the National Academy of Sciences of the United States of America*.

[13] Schembri G, Schober P (2011). Killing two birds with one stone. *The Lancet*.

[14] Rose NR (2002). Mechanisms of autoimmunity. *Seminars in Liver Disease*.

[15] Shimoda S, Nakamura M, Ishibashi H, Hayashida K, Niho Y (1995). HLA DRB4 0101-restricted immunodominant T cell autoepitope of pyruvate dehydrogenase complex in primary biliary cirrhosis: evidence of molecular mimicry in human autoimmune diseases. *Journal of Experimental Medicine*.

[16] van de Water J, Ishibashi H, Coppel RL, Gershwin ME (2001). Molecular mimicry and primary biliary cirrhosis: premises not promises. *Hepatology*.

[17] Regner M, Lambert PH (2001). Autoimmunity through infection or immunization?. *Nature immunology*.

[18] Zhang G, Chen M, Graham D Mouse mammary tumor virus in anti-mitochondrial antibody producing mouse models.

[19] Oertelt S, Lian ZX, Cheng CM (2006). Anti-mitochondrial antibodies and primary biliary cirrhosis in TGF-*β* receptor II dominant-negative mice. *Journal of Immunology*.

[20] Aoki CA, Roifman CM, Lian ZX (2006). IL-2 receptor alpha deficiency and features of primary biliary cirrhosis. *Journal of Autoimmunity*.

[21] Koarada S, Wu Y, Fertig N (2004). Genetic control of autoimmunity: protection from diabetes, but spontaneous autoimmune biliary disease in a nonobese diabetic congenic strain. *Journal of Immunology*.

[22] Massague J (1998). TGF-beta signal transduction. *Annual Review of Biochemistry*.

[23] Gorelik L, Flavell RA (2000). Abrogation of TGF*β* signaling in T cells leads to spontaneous T cell differentiation and autoimmune disease. *Immunity*.

[24] Harada K, Isse K, Tsuneyama K, Ohta H, Nakanuma Y (2003). Accumulating CD57^+^CD3^+^ natural killer T cells are related to intrahepatic bile duct lesions in primary biliary cirrhosis. *Liver International*.

[25] Oertelt S, Ridgway WM, Ansari AA, Coppel RL, Gershwin ME (2007). Murine models of primary biliary cirrhosis: comparisons and contrasts. *Hepatology Research*.

[26] Shindo M, Mullin GE, Braun-Elwert L, Bergasa NV, Jones EA, James SP (1996). Cytokine mRNA expression in the liver of patients with primary biliary cirrhosis (PBC) and chronic hepatitis B (CHB). *Clinical and Experimental Immunology*.

[27] Yasoshima M, Kono N, Sugawara H, Katayanagi K, Harada K, Nakanuma Y (1998). Increased expression of interleukin-6 and tumor necrosis factor-*α* in pathologic biliary epithelial cells: in situ and culture study. *Laboratory Investigation*.

[28] Wakabayashi K, Lian ZX, Moritoki Y (2006). IL-2 receptor *α*
^−/−^ mice and the development of primary biliary cirrhosis. *Hepatology*.

[29] Berg PA, Klein R, Röcken M (1997). Cytokines in primary biliary cirrhosis. *Seminars in Liver Disease*.

[30] Irie J, Wu Y, Wicker LS (2006). NOD.c3c4 congenic mice develop autoimmune biliary disease that serologically and pathogenetically models human primary biliary cirrhosis. *Journal of Experimental Medicine*.

[31] Humbert P, Dupond JL (1988). Multiple autoimmune syndromes (MAS). *Annales de Medecine Interne*.

[32] Gtc KO, Szeto CC, Yeung V, Chow CC, Chan H, Cockram CS (1996). Autoimmune polyglandular syndrome and primary biliary cirrhosis. *British Journal of Clinical Practice*.

[33] Lin JP, Cash JM, Doyle SZ (1998). Familial clustering of rheumatoid arthritis with other autoimmune diseases. *Human Genetics*.

[34] Griffiths MM, Encinas JA, Remmers EF, Kuchroo VK, Wilder RL (1999). Mapping autoimmunity genes. *Current Opinion in Immunology*.

[35] Ueno Y, Moritoki Y, Shimosegawa T, Gershwin ME (2007). Primary biliary cirrhosis: what we know and what we want to know about human PBC and spontaneous PBC mouse models. *Journal of Gastroenterology*.

[36] Nakagome Y, Ueno Y, Kogure T (2007). Autoimmune cholangitis in NOD.c3c4 mice is associated with cholangiocyte-specific Fas antigen deficiency. *Journal of Autoimmunity*.

[37] Munoz S, Ballas S, Norberg R, Maddrey W (1988). Antibodies to human immunodeficiency virus (HIV) in primary biliary cirrhosis. *Gastroenterology*.

[38] Talal N, Dauphinee MJ, Dang H, Alexander SS, Hart DJ, Garry RF (1990). Detection of serum antibodies to retroviral proteins in patients with primary Sjogren’s syndrome (autoimmune exocrinopathy). *Arthritis and Rheumatism*.

[39] Talal N, Garry RF, Schur PH (1990). A conserved idiotype and antibodies to retroviral proteins in systemic lupus erythematosus. *Journal of Clinical Investigation*.

[40] Mason AL, Xu L, Guo L (1998). Detection of retroviral antibodies in primary biliary cirrhosis and other idiopathic biliary disorders. *The Lancet*.

[41] Xu L, Sakalian M, Shen Z, Loss G, Neuberger J, Mason A (2004). Cloning the human betaretrovirus proviral genome from patients with primary biliary cirrhosis. *Hepatology*.

[42] Selmi C, Ross SR, Ansari AA (2004). Lack of immunological or molecular evidence for a role of mouse mammary tumor retrovirus in primary biliary cirrhosis. *Gastroenterology*.

[43] Mason AL, Farr GH, Xu L, Hubscher SG, Neuberger JM (2004). Pilot studies of single and combination antiretroviral therapy in patients with primary biliary cirrhosis. *American Journal of Gastroenterology*.

[44] Mason AL, Lindor KD, Bacon BR, Vincent C, Neuberger JM, Wasilenko ST (2008). Clinical trial: randomized controlled study of zidovudine and lamivudine for patients with primary biliary cirrhosis stabilized on ursodiol. *Alimentary Pharmacology and Therapeutics*.

[45] Fukushima K, Ueno Y, Shimosegawa T (2010). Treatment of primary biliary cirrhosis: a new challenge?. *Hepatology Research*.

[46] Chen M, Graham D, Girgis S (2007). Combination antiretroviral therapy with Combivir attenuates autoimmune biliary disease in the NOD.c3c4 mouse model of primary biliary cirrhosis. *Hepatology*.

[47] Graham D, Chen M, Girgis S, Zhang G, Mason A (2008). Highly active anti-retroviral therapy completely abrogates cholangitis in the NOD.c3c4 mouse model of PBC. *Journal of Hepatology*.

[48] Locke GR, Therneau TM, Ludwig J, Dickson ER, Lindor KD (1996). Time course of histological progression in primary biliary cirrhosis. *Hepatology*.

[49] Nagata S, Golstein P (1995). The Fas death factor. *Science*.

[50] Sakai T, Kimura Y, Inagaki-Ohara K, Kusugami K, Lynch DH, Yoshikai Y (1997). Fas-mediated cytotoxicity by intestinal intraepithelial lymphocytes during acute graft-versus-host disease in mice. *Gastroenterology*.

[51] Ueno Y, Ishii M, Yahagi K (2000). Fas-mediated cholangiopathy in the murine model of graft versus host disease. *Hepatology*.

[52] Hiramatsu N, Hayashi N, Katayama K (1994). Immunohistochemical detection of Fas antigen in liver tissue of patients with chronic hepatitis C. *Hepatology*.

[53] Iwata M, Harada K, Hiramatsu K (2000). Fas ligand expressing mononuclear cells around intrahepatic bile ducts co-express CD68 in primary biliary cirrhosis. *Liver*.

[54] Que FG, Phan VA, Phan VH (1999). Cholangiocarcinomas express Fas ligand and disable the Fas receptor. *Hepatology*.

[55] Chen M, Graham D, Zhang G (2007). Biliary infection with mouse mammary tumor virus in the NOD.c3c4 mouse and other mouse models of primary biliary cirrhosis. *Hepatology*.

